# Measuring ex vivo drug susceptibility in *Plasmodium vivax* isolates from Cambodia

**DOI:** 10.1186/s12936-017-2034-2

**Published:** 2017-09-30

**Authors:** Suwanna Chaorattanakawee, Chanthap Lon, Soklyda Chann, Kheang Heng Thay, Nareth Kong, Yom You, Siratchana Sundrakes, Chatchadaporn Thamnurak, Sorayut Chattrakarn, Chantida Praditpol, Kritsanai Yingyuen, Mariusz Wojnarski, Rekol Huy, Michele D. Spring, Douglas S. Walsh, Jaymin C. Patel, Jessica Lin, Jonathan J. Juliano, Charlotte A. Lanteri, David L. Saunders

**Affiliations:** 1 Department of Immunology and Medicine, Armed Forces Research Institute of Medical Science, Bangkok, Thailand; 2 Department of Parasitology and Entomology, Faculty of Public Health, Mahidol University, Bangkok, Thailand; 3 National Center for Parasitology, Entomology and Malaria Control, Phnom Penh, Cambodia; 4 Division of Infectious Diseases, School of Medicine, University of North Carolina, Chapel Hill, NC USA; 5 US Army Medical Materiel Development Activity, Fort Detrick, Frederick, MD USA

**Keywords:** Drug resistance, *Plasmodium vivax*, Cambodia, Ex vivo assay, *pvmdr1*

## Abstract

**Background:**

While intensive *Plasmodium falciparum* multidrug resistance surveillance continues in Cambodia, relatively little is known about *Plasmodium vivax* drug resistance in Cambodia or elsewhere. To investigate *P. vivax* anti-malarial susceptibility in Cambodia, 76 fresh *P. vivax* isolates collected from Oddar Meanchey (northern Cambodia) in 2013–2015 were assessed for ex vivo drug susceptibility using the microscopy-based schizont maturation test (SMT) and a *Plasmodium* pan-species lactate dehydrogenase (pLDH) ELISA. *P. vivax* multidrug resistance gene 1 (*pvmdr1*) mutations, and copy number were analysed in a subset of isolates.

**Results:**

Ex vivo testing was interpretable in 80% of isolates using the pLDH-ELISA, but only 25% with the SMT. *Plasmodium vivax* drug susceptibility by pLDH-ELISA was directly compared with 58 *P. falciparum* isolates collected from the same locations in 2013–4, tested by histidine-rich protein-2 ELISA. Median pLDH-ELISA IC_50_ of *P. vivax* isolates was significantly lower for dihydroartemisinin (3.4 vs 6.3 nM), artesunate (3.2 vs 5.7 nM), and chloroquine (22.1 vs 103.8 nM) than *P. falciparum* but higher for mefloquine (92 vs 66 nM). There were not significant differences for lumefantrine or doxycycline. Both *P. vivax* and *P. falciparum* had comparable median piperaquine IC_50_ (106.5 vs 123.8 nM), but some *P. falciparum* isolates were able to grow in much higher concentrations above the normal standard range used, attaining up to 100-fold greater IC_50_s than *P. vivax*. A high percentage of *P. vivax* isolates had *pvmdr1* Y976F (78%) and F1076L (83%) mutations but none had *pvmdr1* amplification.

**Conclusion:**

The findings of high *P. vivax* IC_50_ to mefloquine and piperaquine, but not chloroquine, suggest significant drug pressure from drugs used to treat multidrug resistant *P. falciparum* in Cambodia. *Plasmodium vivax* isolates are frequently exposed to mefloquine and piperaquine due to mixed infections and the long elimination half-life of these drugs. Difficulty distinguishing infection due to relapsing hypnozoites *versus* blood-stage recrudescence complicates clinical detection of *P. vivax* resistance, while well-validated molecular markers of chloroquine resistance remain elusive. The pLDH assay may be a useful adjunctive tool for monitoring for emerging drug resistance, though more thorough validation is needed. Given high grade clinical chloroquine resistance observed recently in neighbouring countries, low chloroquine IC_50_ values seen here should not be interpreted as susceptibility in the absence of clinical data. Incorporating pLDH monitoring with therapeutic efficacy studies for individuals with *P. vivax* will help to further validate this field-expedient method.

**Electronic supplementary material:**

The online version of this article (doi:10.1186/s12936-017-2034-2) contains supplementary material, which is available to authorized users.

## Background

Cambodia continues to be at the epicenter of globally emerging multidrug resistant malaria. This has prompted intensive elimination efforts accompanied by surveillance to characterize *Plasmodium falciparum* resistance. Over the past several decades, *P. falciparum* has developed resistance to numerous drugs, particularly in Southeast Asia where high grade treatment failures have been documented [1–4]. Since 2000, the use of artemisinin (ART)-based combination therapy (ACT) as first-line treatment has been implemented in nearly all malaria endemic areas to overcome resistance developing as a result of monotherapy treatments. However, *P. falciparum* resistance to ART emerged in 2006, just a few years after introduction of artesunate–mefloquine (AS–MQ) in Cambodia [5, 6], and was later confirmed on a large scale by intensive multidisciplinary surveillance studies [7]. In 2013, the first high grade clinical failures of dihydroartemisinin–piperaquine (DHA–PPQ) were reported [8]. DHA–PPQ had only recently been introduced as first-line therapy in Cambodia. Reduced in vitro *P. falciparum* susceptibility to PPQ developed on a background of artemisinin resistance here [9, 10], with specific molecular mechanisms elucidated soon thereafter [11, 12].

While intensive surveillance for *P. falciparum* continues in Cambodia, little is known about *Plasmodium vivax* drug resistance. Clinical *P. vivax* resistance is far more difficult to characterize due to difficulties distinguishing true recrudescence of resistant parasites, reinfections with new blood-stage *P. vivax* infections, and relapsing infections by liver stage hypnozoites. Moreover, inability to maintain long-term culture of *P. vivax* parasites prevents reproducible assessments of parasite drug susceptibilities. *Plasmodium vivax* resistance is generally assumed to be less pronounced than *P. falciparum*, as blood-stage *P. vivax* infection remains clinically susceptible to most available anti-malarials. CQ resistant *P. vivax* was first reported in 1989 from Papua New Guinea (PNG) and Papua Indonesia [13–16], where CQ monotherapy remains ineffective. Chloroquine-resistant vivax isolates in this region have been found to harbour polymorphisms in the *pvmdr1* (*P. vivax* multidrug resistance 1) gene, whereas amplification of the gene has been associated with reduced susceptibility to MQ and other drugs in vitro [17–20]. Sporadic cases of CQ failure have since been reported in parts of Southeast Asia and South America [21–24], but there is no clear evidence that these are associated with *pvmdr1* mutations in these regions. Thus, the usefulness of this putative drug resistance marker for detecting emerging resistance remains uncertain.

Artemisinin-based combination therapy is now recommended for use as first-line agent for all malaria in areas of multidrug resistant *P. falciparum*, particularly where clinical failures have been documented [25, 26]. In Cambodia, DHA–PPQ was used as first-line therapy for both vivax and falciparum malaria since 2011 to respond to declining efficacy of CQ for *P. vivax* treatment in specific northern and western provinces where the clinical cure rate had dropped to 80–90%, though cure rates have remained near 100% elsewhere [27–29]. The approach was also implemented to simplify drug administration and overcome diagnostic difficulties. Limited diagnostic capacity makes distinguishing *P. vivax* from *P. falciparum* microscopically challenging, despite overall increases in diagnostic capacity after years of effort. Frequent relapse of latent *P. vivax* following treatment for blood stage *P. falciparum* infections, and high prevalence of difficult to distinguish mixed species infections pose further challenges. While the use of DHA–PPQ to treat CQ-sensitive *P. vivax* is thought to be effective and convenient [26], it may in fact exacerbate resistance to both *P. vivax* and *P. falciparum* [30].

To investigate *P. vivax* anti-malarial susceptibility in Cambodia, fresh *P. vivax* isolates collected from Northern provinces from 2013 to 2015 were assessed for sensitivity to commonly used drugs in short-term culture. Growth inhibition was measured by both the microscopy-based schizont maturation test (SMT) and *Plasmodium* pan-species lactate dehydrogenase (pLDH) ELISA. SMT had been considered the conventional method for *P. vivax* drug testing, but not sensitive for low parasitaemia samples. It is also labour-intensive and interpretation of results is subjective. pLDH ELISA has emerged, offering notable advantages. pLDH has demonstrated higher sensitivity for detection in settings of low *P. vivax* growth rate and very low parasitaemia, improving yields for IC_50_ determination [31]. Results from these assays were then compared with those from concurrently collected *P. falciparum* isolates analysed in both the novel pLDH assay and the previously established HRP-2 assay [32]. *Plasmodium vivax* multidrug resistance gene 1 (*pvmdr1*) mutations and copy number, which have been proposed as candidate markers for drug resistance, were analyzed in a subset of isolates. This is the first report that we are aware of that documents ex vivo *P. vivax* drug susceptibility in Cambodia, and provides baseline data for future surveillance and elimination efforts.

## Methods

### Study site, sample collection and processing

*Plasmodium* isolates were obtained from volunteers with uncomplicated malaria enrolled in an anti-malarial drug resistance surveillance study conducted June 2013–October 2015, in Oddar Meanchey province, northern Cambodia. The study was approved by the Cambodian National Ethics Committee for Health Research (NECHR), and the Walter Reed Army Institute of Research (WRAIR) Institutional Review Board (protocol WR1576). All subjects were ≥ 13 years old without a history of anti-malarial drug use within the past 7 days. Diagnosis of malaria was performed using Giemsa-stained peripheral blood smears, and confirmed later by real-time PCR [9]. A total of 76 *P. vivax* isolates were collected from volunteers with mono *P. vivax* from June 2013 to October 2015, while 58 *P. falciparum* isolates were collected for comparison from those with mono *P. falciparum* infections from January to August 2014. After informed consent, patient venous blood samples were collected in sodium heparin and directly tested fresh for ex vivo drug susceptibility within 6 h of phlebotomy without blood centrifugation, leukocyte depletion or culture adaptation. Additional blood was collected in an EDTA tube for molecular marker analysis for *P. vivax* drug resistance.

**Fig. 1:**
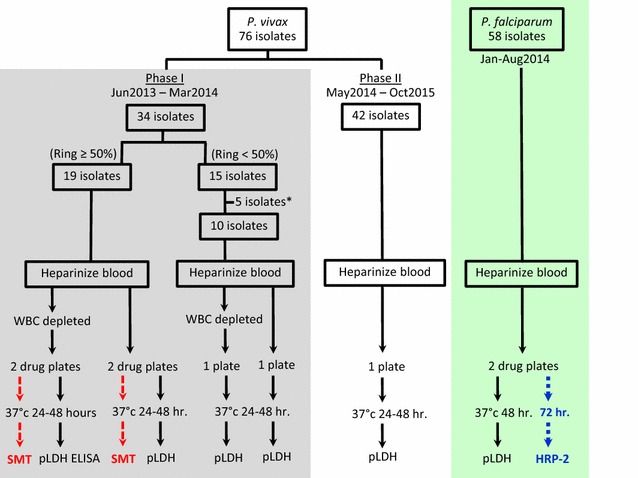
Schematic for ex vivo experiments. *Plasmodium vivax* ex vivo experiments were divided into 2 phases. In Phase I, the schizont maturation test (SMT) was performed concurrently with the pLDH ELISA. Blood samples were processed to deplete white blood cells, and incubated with drugs of interest in parallel with whole blood samples. The SMT was conducted only in samples with ≥ 50% ring stages. *5 isolates with ring stage < 50% in initial samples were not tested for drug susceptibility, but 3 of them had Percoll treatment applied to separate late stage parasites and enrich for early stages for the SMT. In phase II, only pLDH ELISA was performed on whole blood samples. For *P. falciparum* testing, pLDH ELISA was performed concurrently with the HRP-2 ELISA with 48 h incubation times used in the pLDH ELISA to ensure meaningful comparison of *P. vivax* susceptibility, while the previously established 72 h incubation was followed for the HRP-2 method

In the first 29 *P. vivax* isolates, a portion of heparinized blood was centrifuged, and cell pellets including buffy coat were passed through CF11 cellulose (Whatman™, Maidstone, UK) or Plasmodipur filters (EuroProxima, The Netherlands) to remove human white blood cells (WBC). WBC depleted samples were tested for drug susceptibility side by side with whole blood samples. Samples were treated with 45% Percoll in only the first 3 *P. vivax* isolates to separate late stage parasites and enrich for and synchronize at early stages for the schizont maturation test (SMT) [33]. An overall schematic for the experiment is shown in Fig. 1.

### Preparation of dried drug plates

Dried drug-coated plates were prepared using published methods, and tested against the *P. falciparum* W2 reference clone for quality control [32, 34]. All drugs were provided by Chemical Repository of the Walter Reed Army Institute of Research (WRAIR). Briefly, dihydroartemisinin (DHA), artesunate (AS), mefloquine hydrochloride (MQ), quinine sulfate hydrate (QN), chloroquine diphosphate (CQ), and piperaquine phosphate (PPQ) were coated onto 96 well plates. Atovaquone (ATQ), lumefantrine (LUM), doxycycline (DOX) and artemisone (ATM) were added only to later plate lots once the initial methods had been established, and as a result were tested in only 76% (ATQ and LUM), 60% (DOX), and 49% (ATM) of isolates, respectively. Final drug concentrations (after adding samples) ranged from 0.095 to 70 nM for DHA, 0.07 to 52 nM for AS, 0.66 to 482 ng/ml for MQ, 2.18 to 1596 nM for QN, 5.31 to 3877 nM for CQ, 0.9 to 674 nM for PPQ, 0.38 to 273 nM for ATQ, 0.13 to 95 nM for LUM, 285 to 207,943 nM for DOX, and 0.02 to 25 ng/ml for ATM. Starting in 2013, some *P. falciparum* isolates were able to grow in extremely high PPQ concentrations [35]. From 2014 onward, increased PPQ concentration (3.4–53,905 nM) was used in addition to the standard dilutions to ensure accurate inhibitory concentrations could be determined. The top row of each plate served as a drug-free control.

### Immediate ex vivo drug susceptibility assay

At the time of diagnosis (before treatment), fresh *Plasmodium* isolates were tested for sensitivity to anti-malarials by culturing on dried drugs coated plates and measuring growth inhibition. For the *P. vivax* assay, blood samples were adjusted to 2% haematocrit in McCoy’s 5A medium supplemented with 20% AB blood-group serum, and added to dried drug coated plates. Plates were then incubated at 37 °C in a candle jar for 24–48 h, and parasite maturation in drug-free wells was checked by microscopy at 24 h and then every 6 h until 48 h. Parasite growth inhibition over the established concentration ranges was measured by the microscopy-based schizont maturation test (SMT) using the modified WHO microtest [36, 37] for the first 19 *P. vivax* isolates with > 50% early stage parasites in initial samples and *Plasmodium* pan-species lactate dehydrogenase (pLDH) ELISA [31, 38] for all isolates, side by side (Fig. 1). For the SMT, incubation was stopped when ≥ 40% of parasites in drug-free wells had reached the schizont stage. Thick smear examination of samples from each well was performed, and the number of schizonts per 200 asexual stage parasites for each drug concentration was determined. The number of schizonts were plotted against drug concentrations, and IC_50_s were estimated by nonlinear regression analysis using GraphPad Prism version 6.0. For the pLDH-ELISA assay, incubation was stopped when ≥ 40% schizont stages were reached in drug-free wells, or at 48 h if parasite cultures did not reach at least 40% schizonts. Plates were frozen and later thawed for analysis of growth inhibition using the pLDH-ELISA. pLDH optical density (OD) readings were plotted against drug concentrations, and IC_50_s were estimated as described above. Samples with poor growth rates, defined as < 40% schizonts in drug-free wells or a pLDH-OD ratio < 1.7 between no-drug control wells and maximum tested drug concentrations, were excluded from data analysis for SMT or pLDH-ELISA, respectively. A “successful” IC_50_ assay result was defined as achieving a sigmoidal concentration–response when testing serial drug dilutions for at least one of the tested drugs.

For the *P. falciparum* assay, the pLDH-ELISA [38] and the established histidine-rich protein-2 (HRP-2) ELISA [32] was used to simultaneously test field isolates and reference laboratory clones (W2 and 3D7). Culture conditions used in the *P. falciparum* assay were adjusted to 1.5% haematocrit in 0.5% Albumax RPMI 1640. Parasite growth inhibition was assessed by pLDH-ELISA and HRP-2 ELISA after 48 and 72 h incubation, respectively. The same incubation period (48 h) for *P. vivax* and *P. falciparum* were used in the pLDH ELISA to ensure meaningful comparison of susceptibility results. The previously established 72 h incubation was followed for the HRP-2 method. IC_50_s were estimated as described above. Isolates with reduced susceptibility to PPQ capable of growing in maximum drug concentrations could not be interpolated using the standard PPQ dilution range (0.9–674 nM). To accurately estimate IC_50_ dose–response, the curve was replotted by fitting ‘zero-growth’ HRP2 or pLDH OD values at the extrapolated PPQ concentration of 53,905 nM [35, 39].

### *Plasmodium vivax* multi-drug resistance (*pvmdr1*) SNPs and copy number analysis

Genomic DNA was prepared from EDTA-anticoagulated blood using the QIAamp DNA Mini Kit (Qiagen) and used to analyse *pvmdr1* Y976F and F1076L mutations and copy number variation. Multiplex real-time PCR was used to assess *pvmdr1* copy number. The *pvmdr1* (target) and *pv aldolase* (reference) genes were both amplified by RT-PCR, following methods described by Lin et al. [40]. Plasmids containing cloned fragments of *pvmdr1* and *pv aldolase*, developed using the TOPO XL PCR Cloning Kit (Invitrogen, Carlsbad, CA), were used as positive controls in each experiment. Reactions were performed in duplicate. Threshold cycle (Ct) values were used to calculate the relative quantitation of *pvmdr1* copy number by the Pfaffl method [41]. Copy number was determined by rounding to the nearest integer and was considered increased if > 1. For *pvmdr1* Y976F and F1076L analysis, PCR was performed to amplify a 647 bp region of the *pvmdr1* gene covering these SNPs, using the conditions previously described in Lin et al. and amplified products were directly sequenced [40].

### Statistical analysis

Statistical analysis was performed using Graph-Pad Prism version 6.0 (GraphPad Software, Inc, San Diego, CA, USA). Parasite drug susceptibilities were expressed as median IC_50_s for all isolates. Differences in susceptibility between groups were determined using non-parametric Mann–Whitney or Kruskal–Wallis tests. Comparison of IC_50_s attained from pLDH and HRP-2 ELISA were made using Wilcoxon matched pair testing. Assay correlation was evaluated using Spearman’s correlation test. Spearman test was also used to examine the correlation between IC_50_ and ratio of early/mature stage parasites (EM ratio) at start of assay to determine the effect of initial *P. vivax* stage on LDH-ELISA IC_50_ results.

## Results

### Ex vivo *P. vivax* assay development using the schizont maturation test

An overall schematic for the experiment is shown in Fig. 1. Of 76 *P. vivax* isolates collected from Oddar Meanchey Province between June 2013 and October 2015, 71 fresh isolates were tested for ex vivo sensitivity to anti-malarials. Parasite density ranged from 2500 to 38,000 µL^−1^ with variation in the initial parasite stage. Synchronous (≥ 80%) early and mature trophozoite stages were found in 13 and 19 samples, respectively, while the rest included mixed stages. In an effort to improve suitability for the schizont maturation test (SMT) by synchronizing isolates at the early stage, the first 3 isolates were treated with 45% Percoll to separate late stage parasites and enrich for early stage. Although Percoll treatment helped to increase a proportion of early stages in samples, trophozoites remained, leading to failure to reach 80% early stage for all 3 samples. One explanation is that the density of 45% Percoll normally can separate the schizonts and very late trophozoites, leaving earlier trophozoites and ring stages in the samples. In addition, perfect separation efficiency of Percoll density centrifugation method may not be achievable in practice as even if synchronized late stages are found in interphase layer, mixed stage parasites may still be found in the pellet. The effect of white blood cell (WBC) depletion processes on parasite maturation were compared for the first 29 isolates, but far fewer schizonts in drug-free wells were observed in WBC-depleted samples (0–29% schizonts), compared to whole blood samples (0–72% schizont). None of the WBC depleted samples reached the threshold for successful culture for SMT (≥ 40% schizonts in drug-free wells) [37]. Given that neither Percoll treatment nor WBC depletion improved assay conditions, both were abandoned. To examine the effects of WBC on the assay, the pLDH assay was run on whole blood and packed RBC specimens of these 29 *P. vivax* isolates, side by side. Good correlations were found for all drugs tested (ρ = 0.5–0.9, *P* < 0.05), though slightly reduced IC_50_s were found after removing WBC for DHA, AS, and CQ with median difference of = − 0.6, − 0.8, − 6.2, respectively. No significant differences were found for other drugs. Reasonable correlation of results attained from both approaches suggested assay reliability when using whole blood samples.

In ex vivo assays of whole blood samples, erythrocytic *P. vivax* maturation was observed for all but 5 isolates in no-drug control wells over 48 h culture resulting in 2–72% schizonts (median = 21%). However, only 22 of 71 isolates were able to develop to ≥ 40% schizonts. The time to maximum ex vivo schizont growth varied from 24 to 48 h, with a median of 48 h, though it tended to be earlier for cultures with mature trophozoite stages taking only 24–30 h. There was a positive correlation between higher ratios of early/mature trophozoites (EM ratio) and time taken to reach maximum schizont growth (Spearman ρ = 0.63; *P* < 0.001). Of the first 19 *P. vivax* isolates with > 50% early stage parasites in initial samples, SMT yielded an interpretable IC_50_ in only 6 isolates (32% success rate). Since the SMT was found to have fewer interpretable results and was significantly more labour-intensive than pLDH-ELISA, the SMT was not performed in the remaining experiments.

### Comparability of ex vivo *Plasmodium falciparum* susceptibility in the HRP-2 and pLDH ELISA assays

**Fig. 2:**
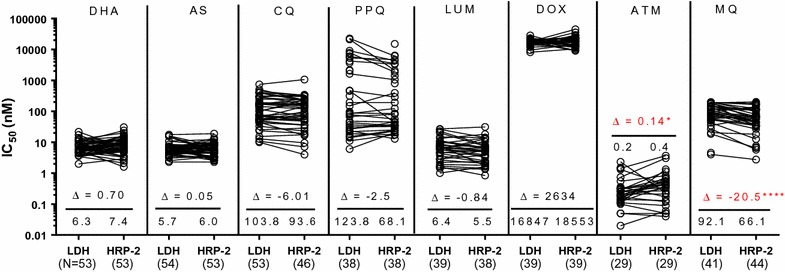
Comparison of *P. falciparum* IC_50_ values attained from pLDH and HRP-2 ELISA. Fresh *P. falciparum* isolates were tested for drug susceptibility using pLDH and HRP-2 ELISA, side by side. Median differences (Δ) are indicated below each pairwise comparison with values from the respective assays for each isolate joined by black lines. Unconnected dots are those where a corresponding value could not be obtained in either the pLDH or HRP-2 ELISA. Median IC_50_ and numbers of evaluable isolates from the respective assays appear above the X-axis, Significant *P*-values from the Wilcoxon pair test are indicated as *(*P* < 0.05), **(*P* < 0.01), ***(*P* < 0.001), ****(*P* < 0.0001)

**Fig. 3:**
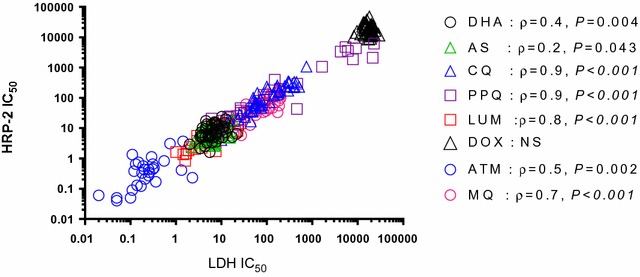
Correlation between *P. falciparum* IC_50_ values attained from pLDH and HRP-2 ELISA. There were moderate to strong correlations between results obtained for *P. falciparum* isolates in the pLDH and HRP-2 ELISAs for all drug tested, with the exception of doxycyline. Significant *P*-values and correlation coefficients (ρ) from Spearman correlation test were indicated. NS indicates not significant based on a *P*-value ≥ 0.05

Given the challenges of the SMT, the pLDH ELISA was evaluated next. In order to first establish the relevance of the pLDH ELISA assay for comparing *P. falciparum* to *P. vivax* resistance profiles, *P. falciparum* IC_50_ results in our routinely used HRP-2 assay and the pLDH ELISA were compared. Figure 2 revealed similarities between *P. falciparum* isolate IC_50_ in the HRP-2 and pLDH ELISA assays for chloroquine (CQ), lumefantrine (LUM), the artemisinins (ARTs), piperaquine (PPQ), and doxycycline (DOX). Wilcoxon pairwise IC_50_ comparisons were significantly higher in the pLDH assay for mefloquine (MQ). Figure 3 illustrates global correlation for *P. falciparum* isolates susceptibility between the 2 assays. There were moderate to strong correlations between results obtained for *P. falciparum* isolates in the pLDH assay and HRP-2 ELISAs for all drugs tested, with the exception of DOX. The latter findings were despite similarities in median values overall.

### Comparison of ex vivo *P. vivax* and *P. falciparum* drug susceptibility in Cambodia 2013–2015 using the pLDH-ELISA method

**Fig. 4:**
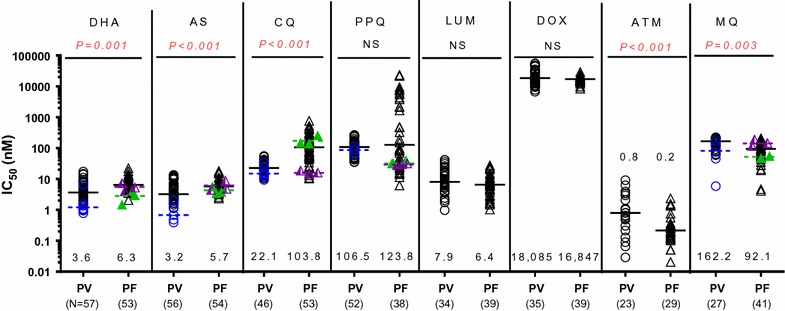
Ex vivo drug susceptibility of *P. vivax* and *P. falciparum* isolates collected from Oddar Meanchey Province (northern Cambodia) during 2013–2015. pLDH-ELISA IC_50_s of fresh *P. vivax* (PV) and *P. falciparum* (PF) isolates against commonly used anti-malarials are presented as black dot plots with black median bars, while SMT results for the only six evaluable PV isolates are indicated in blue. Circle represent PV isolates, while triangles represent PF. Median pLDH-ELISA IC_50_ and numbers of evaluable isolates appear above the X-axis, and the *P*-values for the Mann–Whitney test comparing IC_50_s of *P. vivax* and *P. falciparum* appear at the top of the graph. NS indicates not significant based on a *P*-value ≥ 0.05. pLDH-ELISA IC_50_ of W2 (green) and 3D7 (purple) reference clones obtained from three independent assays are presented in scatter dot plot with bars representing averages

pLDH-ELISA IC_50_s to anti-malarials of Cambodian *P. vivax* isolates collected during 2013–2015 are shown in Fig. 4. Limited SMT data as described above hindered meaningful comparison of results from these 2 assays. pLDH-ELISA IC_50_ results from *P. falciparum* isolates collected in the same area, and from laboratory clones are also presented in Fig. 4 for comparison. IC_50_ results of *P. falciparum* laboratory clones were able to successfully discriminate expected susceptibility profiles for the 3D7 (CQ-sensitive, MQ-resistant) and W2 strains (CQ-resistant, MQ-sensitive). pLDH-ELISA IC_50_s were significantly lower for *P. vivax* than *P. falciparum* clinical isolates for dihydroartemisinin (DHA), artesunate (AS), and CQ, while no differences were found for LUM where both had low IC_50_ or DOX where both had high IC_50_ (Fig. 4). In contrast, pLDH-ELISA IC_50_s of *P. vivax* isolates were significantly higher for artemisone (ATM) and MQ than *P. falciparum* in this assay.

Both *P. vivax* and *P. falciparum* had comparable median PPQ IC_50_ (106.5 vs 123.8 nM respectively), but some *P. falciparum* isolates were able to grow in much higher concentrations above the normal standard curve used (Fig. 4). Although a statistically significant difference was not detected initially when comparing PPQ susceptibility between *P. vivax* and *P. falciparum* based on the pLDH assay, 67% of *P. falciparum* isolates (39 out of 58 total) were found to survive exposure to maximum standard curve PPQ levels (674 nM). Some isolates were found to have few 100-fold greater IC_50_s (470–22,000 nM) using a new testing paradigm with increased maximal PPQ concentrations of 53,905 nM, compared to those obtained in previous years (< 50 nM). In contrast, none of the evaluated *P. vivax* isolates were able to grow in the highest tested PPQ concentration of 674 nM, suggesting greater overall susceptibility compared to *P. falciparum*. *P. vivax* susceptibility to PPQ increased over the period 2013–2015, while there were minor fluctuations or no change in parasite susceptibility to the other drugs tested (Additional file 1).

In contrast to the SMT, pLDH-ELISA was able to determine IC_50_ in 83% of *P. vivax* isolates (58/70) evaluated, though success rate was drug dependent, ranging from 66 to 83% for most drugs. There was < 20% success in achieving sigmoidal IC_50_ curves for quinine (QN) and atovaquone (ATQ). High background optical density (OD) levels in maximally concentrated wells interfered with interpretation. An OD ratio < 1.7 between drug-free control and maximum concentration wells precluded interpretable concentration–response curves. Therefore, QN and ATQ IC_50_ results were excluded from the analysis. Likewise MQ had a lower success rate (39%) compared to other drugs. The high pLDH-OD background interference observed during QN, ATQ, and MQ testing was also observed in *P. falciparum* assays but had less effect on interpretability, and was not detected when *P. falciparum* was tested in the HRP-2 ELISA. The success rate of pLDH-ELISA for *P. falciparum* isolates ranged from 62 to 98% for all drugs tested.

### Effect of initial *P. vivax* stage, assay incubation time and ex vivo growth efficiency on pLDH-ELISA IC_50_ results

To determine the effect of initial *P. vivax* stage on pLDH-ELISA results, the relationship between initial parasite stage and IC_50_ was examined (Table 1). There was a negative correlation between early/mature trophozoite (EM) ratio and IC_50_ for DOX (ρ = − 0.7) with lesser effects observed for DHA, AS and LUM (ρ = − 0.3 to − 0.5). Three to four-fold greater IC_50_ for these drugs were detected in *P. vivax* isolates with synchronous mature trophozoites initially (EM ratio ≤ 0.25) than those with synchronous early stages (EM ratio ≥ 4). However, there was only a borderline correlation between EM ratio and IC_50_ for MQ, and no effect for other drugs.

**Table 1:** Effect of initial *P. vivax* stage on pLDH-ELISA IC_50_ results

Drug	Spearman correlation test			Mann–Whitney U test				*P* value
	for IC_50_ and EM ratio			EM ratio ≥ 4		EM ratio ≤ 0.25		
	N	coefficient	*P*-value	N	median IC_50_	N	median IC_50_	
DHA	57	− 0.34*	0.009*	11	2.6	12	7.5*	0.008*
AS	56	− 0.46*	*<* 0.001*	11	2.9	11	9.4*	*<* 0.001*
CQ	46	− 0.12	0.445	12	23.8	4	28.1	0.716
PPQ	52	− 0.01	0.922	12	104.3	7	99.5	0.612
LUM	34	− 0.42*	0.014*	4	5.6	7	25.4*	0.023*
DOX	35	− 0.70*	*<* 0.001*	5	12,680	9	42,667*	0.003*
ATM	23	− 0.12	0.593	3	0.61	7	2.0	0.137
MQ	27	− *0.39*	0.046*	4	161.2	4	203.7	0.083

* Statistical significance

The influence of assay incubation time on pLDH-ELISA IC_50_ results was examined, and there were noticeable effects when testing DHA, AS, LUM, and DOX. There were significant increases in IC_50_ for these drugs in isolates requiring shorter incubations (24–30 h) compared to longer ones (42–48 h) (Kruskall–Wallis *P* value < 0.001–0.016). To assess the effect of ex vivo growth efficiency on assay results, pLDH-ELISA IC_50_ of isolates in the drug-free control assay reaching ≥ 40% schizonts (the designated threshold for SMT culture success) was compared with results of isolates having less growth efficiency. There were no differences in IC_50_ between the 2 groups for any of the drugs tested (Table 2).

**Table 2:** There was little effect of ex vivo *P. vivax* growth on pLDH-ELISA IC_50_ results

Drug	Maximum number of schizonts				*P* value of Mann–Whitney U test
	< 40% schizont		≥ 40% schizont		
	N	median IC_50_	N	median IC_50_	
DHA	37	3.0	20	4.1	0.108
AS	37	3.0	19	3.5	0.166
CQ	32	22.1	14	22.1	0.981
PPQ	35	108.0	17	105.0	0.513
LUM	23	7.3	11	9.3	0.118
DOX	24	16,021	11	31,326	0.102
ATM	15	0.6	8	1.0	0.175
MQ	19	150.0	8	179.1	0.426

### *Plasmodium vivax* drug resistance markers

*Plasmodium vivax multi*-*drug resistance* (*pvmdr1*) Y976F and F1076L mutations and copy number were analysed for 23 *P. vivax* isolates collected in 2013. Of 23 evaluated isolates, *pvmdr1* Y976F and F1076L mutations were found in 78% (17/23) and 83% (19/23), respectively, composing 74% (17/23) double mutants, 9% (2/23) single F1076L mutants, and 17% (4/23) wild type isolates. None had *pvmdr1* amplification.

## Discussion

This is the first report of ex vivo *P. vivax* drug susceptibility testing of field isolates in Cambodia, providing important baseline data for ongoing resistance surveillance. The focus of containment and elimination efforts in Cambodia to date has been multidrug resistant *P. falciparum* malaria. Relatively little is known about *P. vivax* drug sensitivity due to the inability to culture *P. vivax* long term. The pLDH method bypasses this critical limitation by using fresh isolates, making it potentially practical and informative for measuring ex vivo *P. vivax* resistance. While further validation of the assay is needed, the correlations observed with the IC_50_ results with *P. falciparum,* in both the pLDH and previously established HRP-2 assays, lend support to its utility as a surveillance tool [32]. The *P. falciparum* HRP-2 assay has been carefully standardized in an effort to reliably produce interpretable IC_50_ results in *P. falciparum* field isolates over time [32]. However, it cannot be used in *P. vivax* which does not produce HRP-2. Unfortunately, we were unable to establish the *P. vivax* SMT using previously described methods. Potential explanations for the poor success rate of SMT could include low parasitaemia and drug residue in samples. Nearly 40% of *P. vivax* samples has parasitaemia < 0.1%, and issues of self-medication and unregulated anti-malarial distribution in Cambodia are well documented [42]. Further, WBC depletion process seems to retard *P. vivax* growth leading to failure for SMT. The loss of *P. vivax* and time spent during the WBC filtration process could be possible reasons. However, less effect was observed in pLDH ELISA in which IC_50_ from whole blood, and WBC depleted samples testing were well correlated. This corresponded with the previous finding on *P. falciparum* HRP-2 method, suggesting the reliability of assay on whole blood sample without WBC removing [43]. Overall, in addition to being a field expedient method, the pLDH method was able to reveal some important information about *P. vivax* susceptibility.

With the possible exception of chloroquine, blood stage *P. vivax* is generally thought to have remained susceptible to a wide variety of anti-malarials, though data is limited. Using the pLDH-ELISA, *P. vivax* appeared significantly more susceptible to dihydroartemisinin (DHA), artesunate (AS), and chloroquine (CQ) than *P. falciparum*,but less susceptible to mefloquine (MQ), and artemisone (ATM), and similarly susceptible to lumefantrine (LUM), piperaquine (PPQ) and doxycycline (DOX). It should be noted that although DOX IC_50_ values were in the micromolar range, they were still below previously proposed values for resistance [44]. The present study reveals Cambodian *P. vivax* isolates appear to remain sensitive to CQ while resistance to other anti-malarials may be worse than previously assumed, though the absence of baseline values precludes definitive conclusions. Although previously established, well-controlled pLDH methods were used here to test both *P. vivax* and *P. falciparum*, inter-species difference and assay bias between 2 species cannot be ruled out. Inter-species comparisons require careful interpretation, especially in the absence of baseline data. In addition, mixed parasite stages found in *P. vivax* samples may confound pLDH results for drugs with stage-specific activity. It is possible that the pLDH results of *P. vivax* here represent an average overall susceptibility of mixed parasite stages. As an example, our findings indicated that mixed stage *P. vivax* remained susceptible to CQ, though CQ has specific ring stage activity [37].

The present study brings to light important methodologic considerations for assessing *P. vivax* resistance in vitro. The effect of parasite growth efficiency on *P. vivax* pLDH assay was minimal. There were not significant differences in pLDH-ELISA IC_50_ for most drugs tested between isolates reaching ≥ 40% schizonts and those with less growth. Thus, the pLDH assay had utility even in *P. vivax* isolates failing to reach the 40% schizont target required for the SMT. pLDH-ELISA validity was also confirmed by successful discrimination of known susceptibility profiles for 3D7 (CQ sensitive, MQ-resistant) and W2 (CQ-resistant, MQ-sensitive) *P. falciparum* laboratory strains. When testing *P. falciparum* clinical isolates, pLDH-ELISA was able to detect PPQ resistance at several 100-fold higher IC_50_, corresponding with results from the 72 h HRP-2 ELISA. Comparison with these previously well benchmarked assays further supports use of pLDH-ELISA for *P. vivax* isolate drug susceptibility testing.

Based on our pLDH-ELISA results, stage-specific drug activity on *P. vivax* growth was apparent for DOX and AS, but less pronounced for DHA, LUM and MQ (Table 1). Isolates initially at the trophozoite stage had significantly higher IC_50_s to these drugs than those initially at the ring stage. Specific activity of chloroquine on ring stages, previously described for the schizont maturation test (SMT), was not detected here using the pLDH-ELISA. Comparative *P. vivax* testing at ring and trophozoite stages for the same isolates may confirm stage-specific activity of these drugs. Duration of drug incubation in the SMT is another factor previously reported to influence in vitro drug responses for *P. vivax* [37]. A prior statistical modelling study of SMT dose–response data indicated that only assays with initial ring stage parasitaemia ≥ 65% and a duration ≥ 35 h produce robust IC_50_ values [45]. More data is required to identify the threshold where the association between IC_50_ assay duration and parasite stage composition disappears in the pLDH ELISA.

Comparing findings of the present study to those reported previously for *P. falciparum* and *P. vivax* isolates from the Brazilian Amazon [46, 47] and Indonesia [36, 48, 49] using pLDH ELISA and SMT assays, Cambodian isolates were found to be less susceptible to MQ and PPQ. However, Cambodian and Brazillian *P. vivax* isolates were more sensitive to CQ than in Papua Indonesia where *P. vivax* CQ resistance has emerged. Another ex vivo study reported *P. vivax* CQ resistance in 60% of isolates collected from the Thai-Myanmar border, and higher median IC_50_ than in Cambodia [50]. Although, comparability of the SMT and pLDH ELISA have yet to be formally established, these regional differences are not surprising. Reduced MQ and PPQ susceptibility of Cambodian isolates reflects higher drug pressure in the region from long term use. High grade PPQ resistance recently emerged in *P. falciparum* with resultant effects on sympatric *P. vivax* infection [10, 30]. Chloroquine resistant *P. vivax* infections have been detected in Indonesia since the 1990s and, in 2008, the national treatment guidelines for *P. vivax* were changed to ACT [51]. Approximately 60% of *P. vivax* patients treated with chloroquine experienced a recurrence within 28 days in studies from Malaysia and Vietnam [24, 52]. Yet chloroquine sensitivity was better preserved in studies conducted in Cambodia [29, 44], the Brazilian Amazon [53], Myanmar [54], India [55] and Ethiopia [56].

Piperaquine phosphate IC_50_s of Cambodian *P. vivax* isolates were higher than Indonesia, but the median was similar to those of *P. falciparum* from the same region. However, some Cambodian *P. falciparum* isolates were able to grow in much higher PPQ concentrations with up to 100-fold greater IC_50_s than *P. vivax,* suggesting greater overall susceptibility in *P. vivax* compared to *P. falciparum*. In 2012, AS–MQ was replaced with DHA-PPQ as national first-line treatment for both *P. falciparum* and *P. vivax* [57]. Since reduced ex vivo *P. falciparum* PPQ sensitivity of Cambodian isolates has corresponded with high grade failure of DHA–PPQ treatment in this region [10, 35], it raises a concern that DHA–PPQ may also become less effective for *P. vivax*. Unlike *P. falciparum*, *P. vivax* does not exhibit concomitant artemisinin resistance. In the absence of an established baseline value, it is possible that *P. vivax* has higher intrinsic PPQ IC_50_ in the assay than *P. falciparum*. Further, the decline observed in *P. vivax* IC_50_ after PPQ was introduced in 2013 may have been the result of a decline in overall parasite fitness in response to developing mutations, similar to previous observations in *P. falciparum* [58]. Regardless, clinical correlations are necessary to define the impact of increased PPQ IC_50_s for *P. vivax*. Also to be explored is whether the change in treatment policy for *P. vivax* may have relieved chloroquine pressure. However, ongoing *P. falciparum* chloroquine resistance comparable to previously observed levels [9, 34] argues against this. Resistance in both parasite species would have been expected to subside if exposure had truly been reduced [59]. Definitive demonstration of assay utility will require comparison of ex vivo pLDH-ELISA results with clinical response in well-controlled *P. vivax* field studies. Limited ability to distinguish recrudescence, relapse, and reinfection clinically may confound interpretation.

While molecular data in the present study was limited by convenience sampling, and available resources, it does offer a few useful observations. The mechanism of CQ resistance in *P. vivax* remains unclear although a few studies have suggested associations with *P. vivax* multi-drug resistance gene *(pvmdr1)* Y976F mutation and *pvcrt*-*o* expression [17, 60]. In northern Cambodian isolates, *pvmdr1* Y976F mutants were observed at extremely high frequency yet when tested in the ex vivo assay, the isolates are CQ-sensitive. Accordingly, CQ has retained clinical efficacy as a *P. vivax* rescue agent in trials conducted there over the past several years by the USAMD-AFRIMS [29]. This argues against the usefulness of the Y976F mutation as a CQ resistance marker [17]. No *pvmdr1* amplification was detected in Cambodian isolates, precluding correlation with ex vivo drug susceptibility to chloroquine or other anti-malarials. No attempt to measure stage-specific *pvcrt*-*o* expression in these clinical isolates was made here. Comparing our findings to those reported previously from other regions revealed geographical differences in *pvmdr1* amplification and mutation prevalence. The prevalence of Y976F and F1076L mutations were high in Cambodia, Indonesia, and Papua New Guinea (70–100%) [36, 61, 62], but no mutant was detected in Brazil [47, 60]. Moderate rates of mutation were previously found in Thailand (18–23% for Y976F and 53–61% for F1076L) [50, 62]. Corresponding to previous findings [40], most Cambodian isolates were double mutants (74%), with single F1076L mutants found in < 10%. The prevalence here differed compared to Thailand where a single F1076L mutation was seen in > 60%. No *pvmdr1* amplification was detected in northern Cambodian isolates tested in the present study, despite reduced susceptibility to MQ. It is possible that if more sensitive assays targeting the pvmdr1 breakpoint were used [63], minority clones with *pvmdr1* amplification may have been detected, given the polyclonal nature of vivax infections in this region [64–66]. *Pvmdr1* amplification rates of 4–37% have been observed in other regions of the country [30, 40]. Similarly, a 7–39% amplification rate was reported in Thailand [40, 62]. There was no *pvmdr1* amplification in Indonesia [36, 67] and low prevalence in Brazil (0.9–4%) [67, 68]. The lack or low rate of *pvmdr1* amplification in some areas of Thailand and Cambodia with intense MQ pressure does not support previous evidence associating *pvmdr1* amplification with MQ pressure [67].

Although the pLDH-ELISA IC_50_ assay as described here represents an important first step to assess *P. vivax* drug susceptibility, the following caveats must be considered. The confounding factors of mechanism and speed of drug action must be taken into account when interpreting results obtained using different in vitro methods. High optical density (OD) background interference in wells containing maximal concentrations of some drugs tested caused low growth ratios and failure to achieve sigmoidal IC_50_ curves. This phenomenon was reported previously to depend on the nature of the drug being tested and detection methodology. Relatively higher OD background tends to be observed in non-artemisinin drugs with a more pronounced effect in ELISA-based assays [69, 70]. This may relate to the later onset of action of non-artemisinins in the parasite life cycle, allowing ring stage parasites to continue to produce the proteins being assayed despite high drug concentrations. This may explain paradoxical growth seen at high concentrations of non-artemisinins common in the pLDH-ELISA, but less frequently in the HRP2-ELISA, and not at all in the microscopy-based SMT [69, 70]. This corresponds with the observation of parasite growth in high concentrations of atovaquone, mefloquine, and quinine in the pLDH-ELISA assay for both *P. falciparum* and *P. vivax*, but not in the HRP2 ELISA or SMT. The SMT’s relatively low throughput, and challenges interpreting concentration–response curves for parasite cultures unable to develop to the threshold of ≥ 40% schizonts may limit its usefulness. The assay requires extensive experience on stage differentiation by operators to be effective and replicable. However, as the malaria map continues to shrink in Cambodia, it may be possible to better concentrate the expert microscopy skills needed to adequately perform the SMT in areas of greatest need.

## Conclusion

Overall, the pLDH-ELISA has the potential to be a useful and replicable method to assess ex vivo *P. vivax* resistance in field isolates in Cambodia with reasonable throughput. Results correlated well with those observed for *P. falciparum* in both the pLDH and better established HRP-2 assays. While current malaria elimination efforts are focused on resistant *P. falciparum* malaria, *P. vivax* malaria may ultimately prove more difficult to eliminate due to its ease of transmission, dormancy, and only primaquine being effective at preventing *P. vivax* relapse. Current tools are also inadequate to differentiate relapse and recrudescence from re-infection. Evaluation of ex vivo *P. vivax* susceptibility using field isolates will likely prove a useful part of the armamentarium to monitor for growing resistance and predict risk of treatment failures. Clinical *P. vivax* resistance can be difficult to detect due to difficulty distinguishing relapsing disease from recrudescence and/or reinfection, and the pLDH assay may prove a useful adjunct in this regard. Incorporating ex vivo pLDH monitoring with therapeutic efficacy studies for individuals with *P. vivax* is advised to further validate this field-expedient method.

## Additional file

**Additional file 1.** Ex vivo drug susceptibility of *P. vivax* by years.

## Abbreviations

DHA: dihydroartemisinin
AS: artesunate
MQ: mefloquine hydrochloride
QN: quinine sulfate hydrate
CQ: chloroquine diphosphate
PPQ: piperaquine phosphate
ATQ: atovaquone
LUM: lumefantrine
DOX: doxycycline
ATM: artemisone
ATRs: artemisinins
ACT: artemisinin-based combination therapy
HRP-2: histidine-rich protein 2
pLDH: *Plasmodium* pan-species lactate dehydrogenase
ELISA: enzyme-linked immunosorbent assay
SMT: schizont maturation test
IC_50_: 50% inhibitory concentration
PCR: polymerase chain reaction
OD: optical density
*Pvmdr1*: *Plasmodium vivax* multi-drug resistance 1
Ct: threshold cycle
WHO: World Health Organization
