# Supplementary material for: Measuring ex vivo drug susceptibility in Plasmodium vivax isolates from Cambodia
Source: Malar J. 2017 Sep 30;16:392. doi: 10.1186/s12936-017-2034-2 (PMC5622433; doi:10.1186/s12936-017-2034-2)
Supplement: Supplementary file 1 — Additional file 1. Ex vivo drug susceptibility of P. vivax by years. [file 12936_2017_2034_MOESM1_ESM.docx]

**Additional file 1 *Ex vivo* drug susceptibility of *P. vivax* by years**

| **Drug** | **Median P. vivax IC50 (range), no. of evaluable isolates** | | | **Change** |
| --- | --- | --- | --- | --- |
| **Tested** | **2013** | **2014** | **2015** | **by years^$^** |
| DHA | 3.8 (0.9- 14.4)  N=14 | 3.7 (1.1- 14.3)  N=33 | 3.0 (1.8- 17.0)  N=10 | *P*=0.983 |
| AS | 3.0 (1.1-12.1)  N=14 | 3.3 (1.2- 13.2)  N=33 | 2.9 (2.2-10.5)  N=9 | *P*=0.770 |
| CQ | 18.2 (10.3- 40.4)  N=15 | 27.5 (10.3- 54.3)  N=25 | 24.2 (17.8- 50.7)  N=6 | ***P*=0.009*** |
| PPQ | 136.0 (94-234.1)  N=15 | 83.8 (34.4- 195.0)  N=31 | 59.1 (34.9- 82.6)  N=6 | ***P*<0.001*** |
| LUM | - | 9.1 (1.0-30.2)  N=31 | 3.9 (2.4- 7.3)  N=3 | *P*=0.134 |
| DOX | - | 19,540  (9,214- 5,3918)  N=25 | 14,118  (6,561-45,971)  N=10 | *P*=0.273 |
| ATM | - | 0.5 (0.03-3.4)  N=15 | 1.5 (0.6-2.0)  N=8 | ***P*=0.024*** |
| MQ | 155.5 (136-195)  N=4 | 160.7 (57.8-218.9)  N=20 | 183.9 (111-198)  N=3 | *P*=0.990 |

**^$^** Change in IC_50_s of *P. vivax* among years was tested by the Mann Whitney U test (DHA, AS, CQ, PPQ, MQ) or Wilcoxon Rank Sum test (LUM, DOX, ATM).

* Statistical significance
